# Immune checkpoint inhibitors in advanced and relapsed/refractory Hodgkin lymphoma: current applications and future prospects

**DOI:** 10.3389/fonc.2024.1397053

**Published:** 2024-04-18

**Authors:** Charles J. Milrod, Ari Pelcovits, Thomas A. Ollila

**Affiliations:** Department of Hematology/Oncology, Brown University, Providence, RI, United States

**Keywords:** Hodgkin lymphoma, immune checkpoint inhibitors, biomarkers, survival endpoints, clinical trials

## Abstract

Classic Hodgkin lymphoma (cHL) treatment paradigms are undergoing a shift with the integration of immune checkpoint inhibitors (ICIs) into both first-line and relapsed/refractory (R/R) regimens. In first-line therapy, the synergy between ICIs and chemotherapy may surpass the previous standards of ABVD and BV-AVD established by landmark trials including RATHL and ECHELON-1. In R/R disease, the combination of ICIs with chemotherapy has begun to challenge the paradigm of chemotherapy as a bridge to consolidative autologous stem cell transplantation. The clinical advances heralded by ICI offer unique challenges to management. ICI treatment and the associated inflammatory response can make the traditional timing and modalities of treatment response assessment difficult to interpret. In contrast to ABVD and BV-AVD, pembrolizumab-AVD results in PET2 positivity rates that are higher and less predictive of treatment response even when ultimate outcomes may be superior. This suggests that the predictive value of PET2 may be less reliable in the ICI era, prompting a reevaluation of response assessment strategies. Looking forward, circulating tumor DNA (ctDNA) may be a promising tool in response-adapted therapy. Its potential to complement or even supersede PET scans in predicting response to ICIs represents a critical advancement. The integration of ctDNA analysis holds the promise of refining response-adapted approaches and enhancing precision in therapeutic decision-making for patients with cHL. This review navigates the evolving landscape of cHL therapy, emphasizing the paradigmatic shift brought about by ICIs. This article explores the impact of combining ICIs with chemotherapy in both relapsed/refractory and first-line settings, scrutinizes the challenges posed to response-adapted therapy by ICIs, and highlights the potential role of ctDNA as an adjunct in refining response-adapted strategies for cHL.

## Introduction

Classic Hodgkin lymphoma (cHL) stands apart within the landscape of hematological malignancies because of its distinctive histopathological hallmark, the Reed-Sternberg cell, and the intricate interplay between these cells and the tumor microenvironment. This unique interaction fosters an immunosuppressive milieu that underpins immune evasion, treatment resistance, and the pursuit of innovative therapeutic avenues (1–3). Major advances, from basic and translational science to phase 3 studies, have demonstrated the strong efficacy of immune checkpoint inhibitors (ICIs) in the management of cHL.

Across diverse malignancies, ICIs have remodeled treatment paradigms by disrupting immune checkpoints and reinvigorating T cell-mediated antitumor responses (4). Particularly intriguing is the application of these agents in the realm of cHL, where the high expression of programmed death-ligand 1 (PD-L1) on Reed-Sternberg cells, acting as a crucial modulator of immune suppression, beckons toward a tailored therapeutic strategy involving ICIs (5).

In the first-line advanced setting, the addition of ICIs to a chemotherapy backbone is showing improved progression-free survival compared with the current standard-of-care therapies (6). In the relapsed or refractory setting, the synergy between ICIs and established cytotoxic chemotherapy regimens has demonstrated high response rates and durable responses when used with consolidative autologous hematopoietic stem cell transplantation (autoHSCT) (7–10). Newer regimens are exploring transplant-free options with ICI maintenance therapy, suggesting a potential avenue for shifting the therapeutic landscape away from the conventional standard involving autoHSCT (10).

However, the paradigm of response-adapted therapy, which relies on positron emission tomography (PET) scans for response assessment after cycle 2 (PET2), presents challenges in the context of ICIs. Multiple trials combining ICIs with chemotherapy have indicated that PET2 does not predict treatment response as well as cytotoxic chemotherapy & targeted therapy alone (11). This discrepancy has been attributed to the higher PET2 positive rate observed in the presence of ICIs, which has not been associated with lower progression-free survival (PFS). As an alternative, circulating tumor DNA (ctDNA) analysis has emerged as a more reliable predictor of response, suggesting the potential for integrating ctDNA into risk stratification models (11).

This comprehensive review aims to highlight the contributory pathophysiology of cHL, delve into clinical trials conducted in both first-line advanced and relapsed/refractory settings, and navigate the complexities surrounding the incorporation of ICIs into existing treatment paradigms. By probing the cellular and molecular underpinnings, assessing the evolving therapeutic strategies, and addressing the challenges of response assessment, this review aspires to provide a comprehensive perspective on the revolutionary impact of ICIs in reshaping the landscape of cHL management.

## The role of checkpoint surface molecules in the pathophysiology of cHL

In 1832, Thomas Hodgkin penned a seminal letter entitled “On some Morbid Appearances of the Absorbent Glands and Spleen,” detailing a case series of seven patients (12). This letter is widely regarded as the first recognition and documentation of lymphoma as a distinct disease. Impressively, the cases he described were preserved and later exhibited in museums. In a remarkable testament to their preservation, three of these original samples underwent re-evaluation in 1999 (13). This analysis revealed that one sample was diffuse large B-cell lymphoma, while the remaining two were identified as Hodgkin lymphoma. Although Thomas Hodgkin didn’t claim to have delineated this specific subtype of lymphoma, the eponymous term ‘Hodgkin lymphoma’ was later attributed to him. At the time of his initial description, the differentiation between lymphoma subtypes, particularly the identification of the characteristic Reed-Sternberg cells, was beyond the scope of available medical knowledge and technology.

Reed-Sternberg cells exhibit characteristic cytogenetic alterations in the 9p24.1 locus, resulting in increased expression of PD-L1/2 downstream (5, 14). These alterations include copy gain, amplification, and polysomy, with copy gain being the most common alteration affecting up to 60% of patients with cHL, followed by amplification and polysomy. Notably, amplification is associated with a higher PD-L1/2 score than copy gain and polysomy. Higher levels of PD-L1 correlate with better treatment responses in advanced and relapsed/refractory settings. Furthermore, specific alterations in the 9p24.1 locus, such as amplification, copy gain, and polysomy, have shown distinct associations with treatment outcomes following immune checkpoint inhibitor therapy. After treatment with nivolumab, patients with 9p24.1 amplification had the highest proportion of complete response, followed by copy gain and polysomy (15).

While ICIs are thought to work through CD8+ T-cell activation in solid tumors, Reed-Sternberg cells frequently do not express both major histocompatibility complex (MHC) class I and B-2-microglobulin, which interact with the T cell receptor of cytotoxic CD8+ T cells (16). Therefore, positive treatment outcomes may involve mechanisms of action beyond CD8+ cytotoxic T cell-mediated activity (17, 18).

Reed-Sternberg cells represent about 1-10% of the tumor immune microenvironment, with the other 90-99% of cells being other immune cells. Within the myeloid component of the tumor microenvironment, enrichment of classic dendritic cells, monocytes, and macrophages has been associated with early relapse after treatment (19). In the lymphoid component, CD4+ T cells are often colocalized with Reed-Sternberg cells and PD-L1-expressing macrophages. This has significant clinical implications as higher expression of MHC class II, the receptor for CD4, was associated with longer progression-free survival in patients who relapsed after autoHSCT (16). Furthermore, response to ICI was associated with expansion of CD4, but not CD8, T cell receptor clonal diversity (20). A notable subset of CD4+ T cells, immunosuppressive T-regulatory cells, is present in higher proportions in cHL than in reactive lymph nodes, and these T-regulatory cells strongly express LAG3, a marker of T cell exhaustion (21).

**Figure 1:**
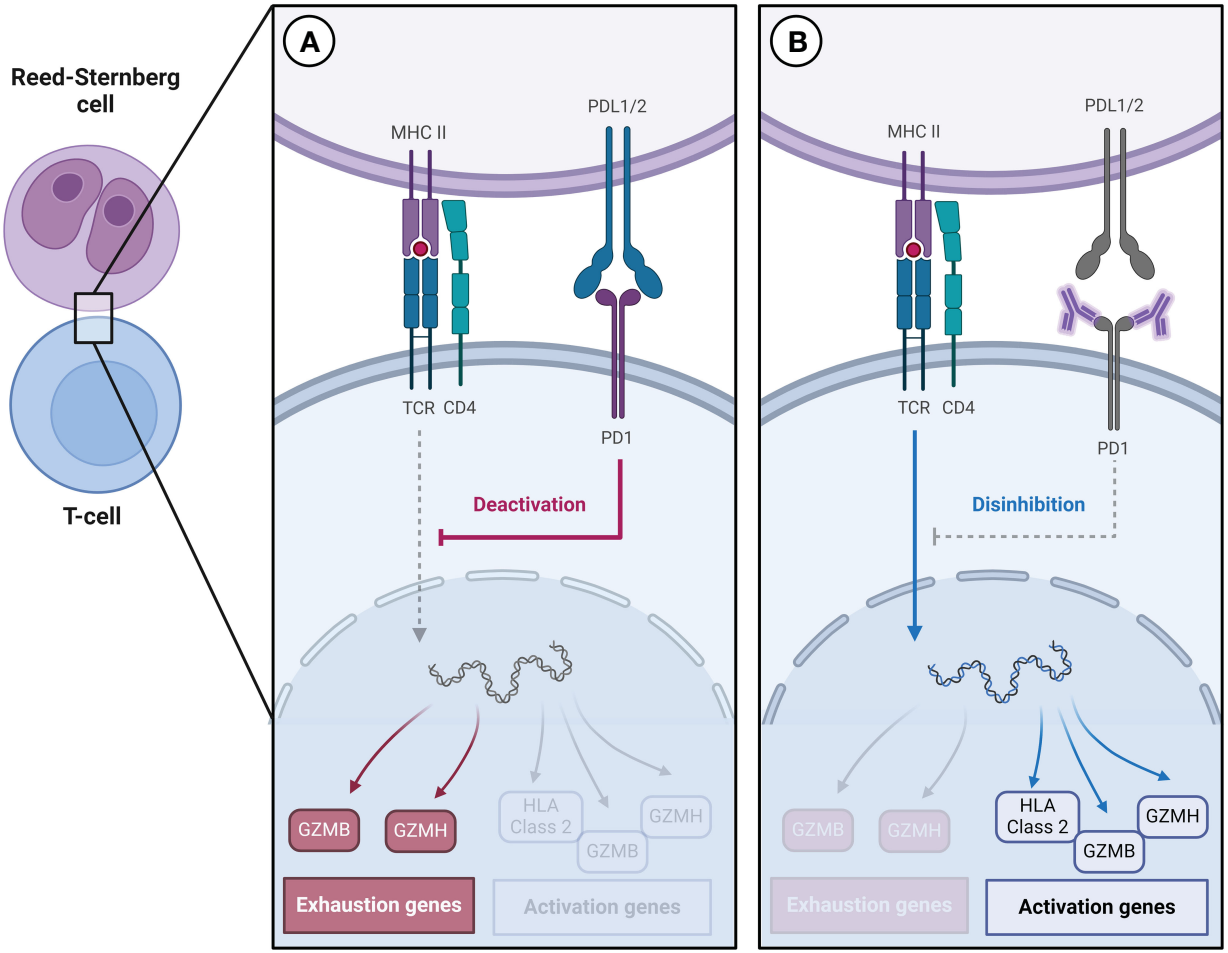
**(A)** Reed-Sternberg cells express high levels of PD-L1/2, which contributes to the increased expression of genes associated with T cell exhaustion. **(B)** Immune checkpoint inhibitors decrease the expression of genes associated with exhaustion and increase the expression of genes associated with activation.

To understand the significance of T cell exhaustion in cHL, insights can be drawn from research conducted on other malignancies. In head and neck cancer, individuals who respond to ICIs tend to have a higher proportion of exhausted T cells before treatment, suggesting that pre-treatment immune features play a fundamental role in treatment response. This phenomenon may be due to ICIs decreasing the expression of exhaustion-related genes, such as TIGIT and TOX, and increasing the expression of cytotoxic genes, including HLA class 2, GZMB, GZMH, and GNLY (**Figure 1**) (22, 23).

In the early untreated cHL setting, similar T cell exhaustion and immunosuppressive tumor microenvironment characteristics were observed. Correlative trials from a German Hodgkin Study Group phase II trial, which randomized participants with early-stage unfavorable Hodgkin lymphoma to receive nivolumab-AVD with or without radiation therapy, demonstrated a rapid reversion of an initially exhausted peripheral blood mononuclear cell phenotype upon treatment (24–26). However, there was no association between disease response and CD8+ cytotoxicity (18, 19). This supports the hypothesis that response to ICI is mediated by reversion of the CD4+ T cell-mediated exhaustion phenotype rather than a CD8+ cytotoxic mechanism.

## Trials in the first-line setting for advanced cHL

In the first-line setting for advanced cHL, ABVD (doxorubicin, bleomycin, vinblastine, and dacarbazine) and escalated BEACOPP (bleomycin, etoposide, doxorubicin, cyclophosphamide, vincristine, procarbazine, and prednisone) have been the standard of care for cHL since the 1990s (27, 28). Studies have shown that eBEACOPP has superior PFS without significant overall survival (OS) benefit at 5-year follow-up, at the expense of increased toxicities (29, 30). However, this PFS advantage diminishes with longer observation time (31, 32). By substituting bleomycin with brentuximab vedotin (BV), BV-AVD and BrECADD (BV, etoposide, cyclophosphamide, doxorubicin, dacarbazine, and dexamethasone) have improved PFS and OS compared with ABVD and eBEACOPP, respectively, and have emerged as an alternative standard of care (33, 34).

Further expanding upon the success of substituting bleomycin with alternative therapies, the SWOG S1826 trial is the first phase 3 trial to investigate ICIs in the first-line setting. This phase 3 trial compared nivolumab-AVD with BV-AVD. The interim analysis demonstrated a 2-year PFS benefit with a hazard ratio of 0.48 (99% CI 0.27-0.87) (6). The OS has not yet been analyzed. Subgroup analysis in older patients shows improved PFS and better tolerability (35). Alternative regimens incorporating ICIs in the first-line setting include the phase 2 trial of pembrolizumab plus AVD, which showed a durable response with a 2-year PFS of 97% (**Table 1**) (11).

**Table 1:** Select trials of recent standard-of-care regimens for untreated advanced cHL.

Regimen arm	eBEACOPP	ABVD	BrECADD	BV-AVD	Nivo-AVD
**Trial**	GHSG HD18 (36–38)	ECHELON-1 (33, 39)	GHSG HD21 (34)	ECHELON-1 (33, 39)	SWOG S1826 (6)
**Years of enrollment**	2008 - 2014	2012 - 2016	2016 - 2020	2012 - 2016	2019 - 2022
**Participant characteristics**	18-60 years Stage 3-4 cHL	_18 years old Stage 3-4 cHL	18-60 years Stage 3-4 cHL	_18 years old Stage 3-4 cHL	>12 years old Stage 3-4 cHL
**PFS**	1-year: — 3-year: 91.4% 5-year: 89.4%	1-year: — 3-year: 76.0% 5-year: 75.3%	1-year: — 3-year: 94.9% 5-year: —	1-year: — 3-year: 83.1% 5-year: 82.2%	1-year: 94% 3-year: — 5-year: —
**OS**	5-year: 95.6%	6-year: 89.4%	3-year: 98.5%	6-year: 93.9%	Not yet analyzed

For patients who are ineligible for ABVD or BV-AVD because of frailty or age, the Niviniho and ACCRU trials investigated alternative ICI-based regimens. Both studies had non-durable responses with median PFS of 9 months with nivolumab plus vinblastine augmentation and 18 months with BV-nivolumab, respectively (**Table 2**) (40, 41). These responses were similar to those seen with nivolumab and pembrolizumab monotherapy (42, 43). This may be due to the included participants being recruited from a more frail population, but also highlights the challenges in current treatment approaches for those who cannot receive chemotherapy.

**Table 2:** Select trials of ICI-based regimens for untreated advanced cHL.

Regimen arm	Nivo +/- vinblastine	BV-nivo	Pembro-AVD	Nivo-AVD
**Trial**	Niviniho (40)	ACCRU (41)	NCT03331341 (11)	SWOG S1826 (6)
**Participants #**	56	46	29	489
**Participant characteristics**	>60 years old Stage 1-4 ECOG 0-2 Considered unsuitable for standard chemotherapy because of comorbidities evaluated by Cumulative Illness Rating Scale (CIRS) score ≥6	>60 years old Stage 1-4 ECOG 0-2 Considered unsuitable for standard chemotherapy because of heart, lung, or kidney function or declined chemotherapy	>18 years old Stage 1-4 ECOG 0 or 1	>12 years old Stage 3-4 Zubrod score 0-2
**ORR**	51.9%	64%	100%	—
**CR**	16%	52%	90%	—
**PFS**	Median: 9 months	Median: 18.3 months	Median: not reached 2-year PFS: 97%	Median: not reached 1-year PFS: 94%

## Trials in the relapsed/refractory setting

ICIs were initially explored as potential treatments for cHL in the refractory/relapsed setting. Trials of pembrolizumab (Keynote 087) and nivolumab (Checkmate 205) monotherapy enrolled heavily pre-treated participants, with a median number of four previous therapies (42, 43). These trials demonstrated an overall response rate of 71.4% and 71.2%, complete response rates of 9% and 27.6%, and a median duration of response of 16.6 and 17 months, respectively. Pembrolizumab was further explored in a phase 3 trial (Keynote 204), with similar results (**Table 3**) (44).

**Table 3:** Select trials of single-agent ICIs for R/R cHL.

Regimen arm	Pembrolizumab	Pembrolizumab	Nivolumab	Tislelizumab
**Trial**	Keynote 087 (42)	Keynote 204 (44)	Checkmate 205 (43)	NCT03209973 (45)
**Median follow-up**	5 years	2 years	5 years	33.8 months
**Participants #**	210	151	243	70
**Participant characteristics**	Median number of previous therapies: 4 Relapsed after autoSCT: 71%	Median number of previous therapies: 2 Relapsed after autoSCT: 37%	Median number of previous therapies: 4 Relapsed after autoSCT: 100%	Median number of previous therapies: 3 Relapsed after autoSCT: 18.6%
**ORR**	71.4%	65.6%	71.2%	87.1%
**CR**	27.6%	25%	21.4%	67.1%
**DOR**	16.6 months	20.7 months	18.2 months	31.3 months

An alternative ICI, tislelizumab, was designed to overcome one of the mechanisms behind PD-PD-L1 pathway blockade resistance, antibody-dependent cellular phagocytosis, by minimizing binding to the cellular receptor on macrophages (46–48). In the phase 2 trial of tislelizumab monotherapy, the results were notable for an overall response rate of 87.1%, complete response rate of 62.9%, and median duration of response of 31.3 months, as detailed in **Table 1** (45). This divergence in outcomes may be due to tislelizumab itself, but alternative reasons include differences in patient characteristics or the criteria employed for tumor assessment. Notably, the trial participants represented a group that had received less prior treatment, with a majority being ineligible for autologous hematopoietic stem cell transplantation (autoHSCT) rather than having experienced progression after autoHSCT. Additionally, it is worth noting that tumor assessment in this trial followed the Lugano criteria, a departure from the 2007 International Working Group Revised Response Criteria for Malignant Lymphomas (IWG 2007) employed in Keynote 087, Keynote 204, and Checkmate 205 (42–45).

In addition to the high rates of response for ICI monotherapy, their low rate of adverse effects and potential chemotherapy-sensitizing properties make them ideal agents for combination regimens (49). The current treatment paradigm for refractory/relapsed cHL involves a combination of cytotoxic chemotherapy with or without ICI, followed by curative-intent autoHSCT (50). Pembrolizumab-GVD and nivolumab-ICE therapy are response-adapted, with 2 additional cycles of Pembro-GVD or NICE given if not in CR at response assessment, and have an excellent 2-year PFS estimates of 100% and 94% (7, 8). There is growing evidence that ICIs sensitize cHL to further therapy, including autoHSCT. In a multicenter retrospective cohort study, patients treated with ICI-based salvage regimens prior to autoHSCT exhibited significantly improved 2-year PFS rates of 98%, compared to 68.8% for those receiving non-ICI-based salvage regimens (51). Other significant clinical outcomes include improved overall response rate and duration of response to subsequent chemotherapy, targeted therapy, transplant conditioning, and chimeric antigen receptor T cell therapy (52).

For those not eligible for autoHSCT, there are alternative regimens. Tislelizumab plus gemcitabine and oxaliplatin (T-GemOx), uses 2 years of maintenance tislelizumab instead of consolidation with autoHSCT. Although further investigation is needed, the trial reported a 100% overall response rate, with only 2 out of 36 participants experiencing progressive disease at the 2-year follow-up, whereas the rest achieved and maintained complete responses (10). Before this trial, durable responses in the relapsed/refractory setting have not been seen in regimens that were not intended as a bridge to autoHSCT (**Table 4**). Brentuximab vedotin plus nivolumab is a chemotherapy-free option that is not response adapted for four cycles and ends with a consolidation autoHSCT. The final analysis of this trial compared all participants versus those who ultimately underwent autoHSCT and demonstrated a 36-month PFS estimate of 77% and 91% respectively (9).

**Table 4:** Select phase 2 trials of ICI-based regimens for R/R cHL.

Regimen arm	Pembro-GVD (2, 39)	NICE (8)	BV-Nivo (9)	T-GemOx (10)	Pembro-vori (53)
**Participants #**	39	42	93	30	32
**Participant characteristics**	Median lines of therapy: 1 Eligible for autoSCT	Median lines of therapy: 1 Eligible for autoSCT	Median lines of therapy: 1.2 Eligible for autoSCT	Median lines of therapy: 1 Ineligible or relapsed after autoSCT	Median lines of therapy: 3 Ineligible for autoSCT Prior anti-PD1 exposure allowed
**Consolidative plan**	AutoSCT	AutoSCT	AutoSCT	Two years of maintenance tislelizumab	No universal consolidative therapy 4/32 patients went to consolidative autoHSCT 3/32 patients went to consolidative alloHSCT
**ORR**	100%	93%	85%	100%	73%
**CR**	95%	91%	67%	96.7%	33%
**PFS**	13 months: 100%	24 months: 72% (all patients), 94% (bridged to autoSCT)	36 months: 77%	12 months: 96%	12 months: 52%

Alternative non-chemotherapy backbones explored in combination with ICIs include the HDAC inhibitor vorinostat. While only having modest activity in R/R cHL as monotherapy, vorinostat is an epigenetic modifying agent that enhances MHC class 1 expression, increases antigen presentation, and increases T cell infiltration to the tumor microenvironment (54). The first trial to investigate pembrolizumab and vorinostat demonstrated an overall response rate of 73% and a complete response rate of 33%. Notably, 56% of PD-1-inhibitor refractory patients responded (53).

## Assessment of response to ICI-based therapy

Patient stratification and outcome prediction play critical roles in guiding decisions regarding treatment duration, intensity, and selection of cancer-directed therapy. Initially, the international prognostic score (IPS) was the foremost predictor of PFS in cHL. This was achieved by considering factors such as white blood cell count, acute phase reactants, patient characteristics, and disease staging (55). However, over time, a more powerful predictor of treatment outcomes has emerged in the form of interim PET scans for response assessment after cycle 2 (PET2) (56, 57). The advent of PET2 response-adapted therapy has led to a diminished predictive value of IPS for treatment response. To enhance prognostication, new variables, including age and absolute lymphocyte count, have been incorporated into the clinical prediction model known as A-HIPI (57–59). Much like the IPS before it, the relevance of PET2 response-adapted therapy requires re-evaluation in the context of evolving treatment paradigms.

Multiple trials combining ICIs with chemotherapy have shown that PET2 may underestimate the treatment response. This discrepancy has been attributed to the higher PET2 positive rate observed in the presence of ICIs, which has not been associated with lower PFS. In the phase 2 trial of pembrolizumab-AVD, participants received 4 to 6 cycles of therapy based on the results of a PET scan after cycle two (PET2). The PET2 positive rate in this trial was approximately 43%, which is higher than previous trials without ICIs, with rates ranging from 16% to 20% (11, 33, 60–63). Among those with PET2 positive disease, the PFS for participants treated with pembro-AVD was significantly higher than those treated with ABVD and BV-AVD in the RATHL and ECHELON-1 trials, respectively (**Table 5**) (11, 33, 64). The potential for falsely high Deauville scores in early PET2 evaluations during ICI treatments calls for a reexamination of the use of PET2 in response-adapted therapeutic strategies.

**Table 5:** PET-response rates across select trials.

	GITIL/FIL HD 0607 (60)	SWOG S0816 (61)	HD0801 (62)	ECHELON-1 (33)	RATHL (63)	Pembro-AVD (11)
**Regimen**	ABVD	ABVD	ABVD	BV-AVD	ABVD	Pembro-AVD
**PET2+ rate**	19%	18%	20%	11%	16%	43%
**PFS for PET2+**	3 years: 60%	5 years: 66%	2 years: 74%	6 years: 61%	3 years: 67.5%	2 years: 97%

Circulating tumor DNA (ctDNA) analysis has recently emerged as a promising tool for predicting treatment response. Interestingly, despite the typically low abundance of Reed-Sternberg cells in tissue-based pathology, ctDNA levels in peripheral blood are relatively high, even exceeding those found in tumor tissue (65–68). Historically, the accuracy and effectiveness of ctDNA assessment were hindered by high error rates and challenges in genome recovery. However, these limitations have been mitigated with the advent of PhasED-Seq technology (69). This approach enhances accuracy by detecting multiple single nucleotide variants on the same DNA strand, thereby reducing the error rate compared to methods that identify single nucleotide variants individually. Furthermore, PhasED-Seq requires only one strand of DNA for detection, unlike previous techniques that necessitated both strands, significantly improving the efficiency of genome recovery and making ctDNA analysis a more viable and reliable method for assessing treatment response. In clinical practice, ctDNA was a better predictor of response in the trial investigating pembro-AVD, as the one patient who relapsed had a negative PET at cycle two but did not clear circulating tumor DNA. Importantly, no patient who cleared circulating tumor DNA has relapsed in this trial (11).

## Conclusion

The decision to utilize ICIs, both in first-line and R/R settings, necessitates a nuanced understanding of patient-specific factors and the broader implications of introducing these therapies into standard practice. This includes considerations of potential autoimmune conditions, treatment history, candidacy for aggressive chemotherapy, and the evolving landscape of biomarker-driven therapy selection. Furthermore, as ICIs redefine treatment benchmarks, there’s a pressing need to recalibrate our response assessment tools, moving beyond traditional metrics like PET2 to more predictive measures such as ctDNA, which may offer a more accurate gauge of treatment efficacy in the era of immunotherapy.

In light of the data presented from the SWOG S1826 trial and other pivotal studies, the incorporation of Nivo-AVD in the first-line treatment of advanced cHL signifies a potential paradigm shift. The trial’s demonstration of a significant improvement in PFS over recently defined benchmark regimen BV-AVD positions Nivo-AVD as an appealing option for upfront therapy. However, the consensus to broadly adopt Nivo-AVD as a standard practice should hinge on the forthcoming OS data. If OS benefits align with the PFS improvements observed, Nivo-AVD should be strongly considered as the new standard for first-line therapy in advanced cHL for most patients.

In the context of R/R cHL, the compelling efficacy of ICIs underscores their fundamental role in this setting. Given their capacity to induce durable responses, especially when integrated with strategies like autoHSCT for eligible patients, ICIs should be regarded as a cornerstone of therapy for R/R cHL. For patients ineligible for transplantation, emerging treatments like T-GemOx, followed by maintenance ICI therapy, offer promising alternatives that may be obviate the need for autoHSCT. This approach not only broadens the therapeutic landscape and potential for cure in less fit or older patients, but also emphasizes the need to revisit the dogma of consolidative autoHSCT in this new treatment era. Unexplored areas of research include the role of ICIs in the R/R after first-line ICI exposure.

The integration of ICIs into cHL treatment signifies more than just an advancement in therapeutic options; it serves as a call to action for the hematological community. It compels us to become proficient in managing a therapeutic agent that, while commonplace among our colleagues treating solid tumors, has seldom been used in hematology. This transition not only necessitates a reevaluation of our standard response assessments but also holds promises to enhance patient outcomes. Moreover, it challenges us to deepen our understanding of cHL biology, optimize treatment strategies, and elevate the standard of care. This evolution marks a significant shift, emphasizing the need for continuous education and adaptation in hematology.

## Author contributions

CM: Conceptualization, Investigation, Writing – original draft, Writing – review & editing. AP: Conceptualization, Writing – review & editing. TO: Conceptualization, Writing – review & editing.

## References

[1] Liu WR Shipp MA. Signaling pathways and immune evasion mechanisms in classical hodgkin lymphoma. Blood (2017) 130(21):2265–70. doi: 10.1182/blood-2017-06-781989 PMC5701523 29167175

[2] Brice P de Kerviler E Friedberg JW. Classical hodgkin lymphoma. Lancet Lond Engl (2021) 398(10310):1518–27. doi: 10.1016/S0140-6736(20)32207-8 33493434

[3] Steidl C Connors JM Gascoyne RD. Molecular pathogenesis of hodgkin’s lymphoma: increasing evidence of the importance of the microenvironment. J Clin Oncol Off J Am Soc Clin Oncol (2011) 29(14):1812–26. doi: 10.1200/JCO.2010.32.8401 21483001

[4] Shiravand Y Khodadadi F Kashani SMA. Immune checkpoint inhibitors in cancer therapy. Curr Oncol Tor Ont (2022) 29(5):3044–60. doi: 10.3390/curroncol29050247 PMC9139602 35621637

[5] Green MR Monti S Rodig SJ. Integrative analysis reveals selective 9p24.1 amplification, increased PD-1 ligand expression, and further induction *via* JAK2 in nodular sclerosing hodgkin lymphoma and primary mediastinal large b-cell lymphoma. Blood (2010) 116(17):3268–77. doi: 10.1182/blood-2010-05-282780 PMC2995356 20628145

[6] Herrera AF LeBlanc ML Castellino SM. SWOG S1826, a randomized study of nivolumab(N)-AVD versus brentuximab vedotin(BV)-AVD in advanced stage (AS) classic hodgkin lymphoma (HL). J Clin Oncol (2023) 41(17_suppl):LBA4–4. doi: 10.1200/JCO.2023.41.17_suppl.LBA4

[7] Moskowitz AJ Shah G Schöder H. Phase II trial of pembrolizumab plus gemcitabine, vinorelbine, and liposomal doxorubicin as second-line therapy for relapsed or refractory classical hodgkin lymphoma. J Clin Oncol Off J Am Soc Clin Oncol (2021) 39(28):3109–17. doi: 10.1200/JCO.21.01056 PMC9851707 34170745

[8] Mei MG Lee HJ Palmer JM. Response-adapted anti-PD-1-based salvage therapy for hodgkin lymphoma with nivolumab alone or in combination with ICE. Blood (2022) 139(25):3605–16. doi: 10.1182/blood.2022015423 PMC9227101 35316328

[9] Advani RH Moskowitz AJ Bartlett NL. Brentuximab vedotin in combination with nivolumab in relapsed or refractory hodgkin lymphoma: 3-year study results. Blood (2021) 138(6):427–38. doi: 10.1182/blood.2020009178 PMC12684812 33827139

[10] Ding K Liu H Ma J. Tislelizumab with gemcitabine and oxaliplatin in patients with relapsed or refractory classic hodgkin lymphoma: a multicenter phase II trial. Haematologica (2023) 108(8):2146–54. doi: 10.3324/haematol.2022.282266 PMC10388287 36700408

[11] Lynch RC Ujjani CS Poh C. Concurrent pembrolizumab with AVD for untreated classic hodgkin lymphoma. Blood (2023) 141(21):2576–86. 10.1182/blood.2022019254 PMC10273164 36913694

[12] Hodgkin null. On some morbid appearances of the absorbent glands and spleen. Medico-Chir. Trans (1832) 17:68–114. 10.1177/095952873201700106 PMC2116706 20895597

[13] Poston RN. A new look at the original cases of hodgkin’s disease. Cancer Treat Rev (1999) 25(3):151–5. doi: 10.1053/ctrv.1998.9997 10425256

[14] Roemer MGM Advani RH Ligon AH. PD-L1 and PD-L2 genetic alterations define classical hodgkin lymphoma and predict outcome. J Clin Oncol Off J Am Soc Clin Oncol (2016) 34(23):2690–7. doi: 10.1200/JCO.2016.66.4482 PMC5019753 27069084

[15] Roemer MGM Redd RA Cader FZ. Major histocompatibility complex class II and programmed death ligand 1 expression predict outcome after programmed death 1 blockade in classic hodgkin lymphoma. J Clin Oncol Off J Am Soc Clin Oncol (2018) 36(10):942–50. doi: 10.1200/JCO.2017.77.3994 PMC5877802 29394125

[16] Carey CD Gusenleitner D Lipschitz M. Topological analysis reveals a PD-L1-associated microenvironmental niche for reed-sternberg cells in hodgkin lymphoma. Blood (2017) 130(22):2420–30. doi: 10.1182/blood-2017-03-770719 PMC5766840 28893733

[17] Jalali S Price-Troska T Bothun C. Reverse signaling *via* PD-L1 supports malignant cell growth and survival in classical hodgkin lymphoma. Blood Cancer J (2019) 9(3):22. doi: 10.1038/s41408-019-0185-9 30783096 PMC6381098

[18] Reinke S Bröckelmann PJ Iaccarino I. Tumor and microenvironment response but no cytotoxic t-cell activation in classic hodgkin lymphoma treated with anti-PD1. Blood (2020) 136(25):2851–63. doi: 10.1182/blood.2020008553 33113552

[19] Stewart BJ Fergie M Young MD. Spatial and molecular profiling of the mononuclear phagocyte network in classic hodgkin lymphoma. Blood (2023) 141(19):2343–58. doi: 10.1182/blood.2022015575 36758207

[20] Cader FZ Hu X Goh WL. A peripheral immune signature of responsiveness to PD-1 blockade in patients with classical hodgkin lymphoma. Nat Med (2020) 26(9):1468–79. doi: 10.1038/s41591-020-1006-1 PMC9673009 32778827

[21] Aoki T Chong LC Takata K. Single-cell transcriptome analysis reveals disease-defining t-cell subsets in the tumor microenvironment of classic hodgkin lymphoma. Cancer Discovery (2020) 10(3):406–21. doi: 10.1158/2159-8290.CD-19-0680 31857391

[22] Oliveira G Wu CJ. Dynamics and specificities of t cells in cancer immunotherapy. Nat Rev Cancer (2023) 23(5):295–316. doi: 10.1038/s41568-023-00560-y 37046001 PMC10773171

[23] Wu C. CTSI distinguished speakers series | catherine wu. (2023).

[24] Bröckelmann PJ Bühnen I Meissner J. Nivolumab and doxorubicin, vinblastine, and dacarbazine in early-stage unfavorable hodgkin lymphoma: Final analysis of the randomized german hodgkin study group phase II NIVAHL trial. J Clin Oncol Off J Am Soc Clin Oncol (2023) 41(6):1193–9. doi: 10.1200/JCO.22.02355 36508302

[25] Voltin C-A Mettler J van Heek L. Early response to first-line anti-PD-1 treatment in hodgkin lymphoma: A PET-based analysis from the prospective, randomized phase II NIVAHL trial. Clin Cancer Res Off J Am Assoc Cancer Res (2021) 27(2):402–7. doi: 10.1158/1078-0432.CCR-20-3303 33122344

[26] Garcia-Marquez MA Thelen M Reinke S. Reverted exhaustion phenotype of circulating lymphocytes as immune correlate of anti-PD1 first-line treatment in hodgkin lymphoma. Leukemia (2022) 36(3):760–71. doi: 10.1038/s41375-021-01421-z PMC8885413 34584203

[27] Reinke S Bröckelmann PJ Iaccarino I. Tumor and microenvironment response but no cytotoxic t-cell activation in classic hodgkin lymphoma treated with anti-PD1. Blood (2020) 136(25):2851–63. doi: 10.1182/blood.2020008553 33113552

[28] Diehl V Franklin J Hasenclever D. BEACOPP, a new dose-escalated and accelerated regimen, is at least as effective as COPP/ABVD in patients with advanced-stage hodgkin’s lymphoma: interim report from a trial of the german hodgkin’s lymphoma study group. J Clin Oncol Off J Am Soc Clin Oncol (1998) 16(12):3810–21. doi: 10.1200/JCO.1998.16.12.3810 9850026

[29] Santoro A Bonadonna G Valagussa P. Long-term results of combined chemotherapy-radiotherapy approach in hodgkin’s disease: superiority of ABVD plus radiotherapy versus MOPP plus radiotherapy. J Clin Oncol Off J Am Soc Clin Oncol (1987) 5(1):27–37. doi: 10.1200/JCO.1987.5.1.27 2433409

[30] Federico M Luminari S Iannitto E. ABVD compared with BEACOPP compared with CEC for the initial treatment of patients with advanced hodgkin’s lymphoma: results from the HD2000 gruppo italiano per lo studio dei linfomi trial. J Clin Oncol Off J Am Soc Clin Oncol (2009) 27(5):805–11. doi: 10.1200/JCO.2008.17.0910 19124807

[31] Mounier N Brice P Bologna S. ABVD (8 cycles) versus BEACOPP (4 escalated cycles ≥ 4 baseline): final results in stage III-IV low-risk hodgkin lymphoma (IPS 0-2) of the LYSA H34 randomized trial. Ann Oncol Off J Eur Soc Med Oncol (2014) 25(8):1622–8. doi: 10.1093/annonc/mdu189 24827123

[32] Viviani S Zinzani PL Rambaldi A. ABVD versus BEACOPP for hodgkin’s lymphoma when high-dose salvage is planned. N Engl J Med (2011) 365(3):203–12. doi: 10.1056/NEJMoa1100340 21774708

[33] Merli F Luminari S Gobbi PG. Long-term results of the HD2000 trial comparing ABVD versus BEACOPP versus COPP-EBV-CAD in untreated patients with advanced hodgkin lymphoma: A study by fondazione italiana linfomi. J Clin Oncol Off J Am Soc Clin Oncol (2016) 34(11):1175–81. doi: 10.1200/JCO.2015.62.4817 26712220

[34] Ansell SM Radford J Connors JM. Overall survival with brentuximab vedotin in stage III or IV hodgkin’s lymphoma. N Engl J Med (2022) 387(4):310–20. doi: 10.1056/NEJMoa2206125 35830649

[35] Borchmann P Moccia AA Greil R. BRECADD IS NON-INFERIOR TO EBEACOPP IN PATIENTS WITH ADVANCED STAGE CLASSICAL HODGKIN LYMPHOMA: EFFICACY RESULTS OF THE GHSG PHASE III HD21 TRIAL. Hematol Oncol (2023) 41(S2):881–2. doi: 10.1002/hon.3196_LBA5

[36] Rutherford SC Li H Herrera AF. Nivolumab-AVD is better tolerated and improves progression-free survival compared to bv-AVD in older patients (Aged ≥60 years) with advanced stage hodgkin lymphoma enrolled on SWOG S1826. Blood (2023) 142(Supplement 1):181–1. doi: 10.1182/blood-2023-180114

[37] Lazarovici J Amorim S Bouabdallah K. Nivolumab first-line therapy for elderly, frail hodgkin lymphoma patients: Niviniho, a lysa phase II study. Blood (2021) 138(Supplement 1):232–2. doi: 10.1182/blood-2021-147863

[38] Cheson BD Bartlett NL LaPlant B. Brentuximab vedotin plus nivolumab as first-line therapy in older or chemotherapy-ineligible patients with hodgkin lymphoma (ACCRU): a multicentre, single-arm, phase 2 trial. Lancet Haematol (2020) 7(11):e808–15. doi: 10.1016/S2352-3026(20)30275-1 33010817

[39] Armand P Zinzani PL Lee HJ. Five-year follow-up of KEYNOTE-087: pembrolizumab monotherapy for relapsed/refractory classical hodgkin lymphoma. Blood (2023) 142(10):878–86. doi: 10.1182/blood.2022019386 PMC10624931 37319435

[40] Ansell SM Bröckelmann PJ von Keudell G. Nivolumab for relapsed/refractory classical hodgkin lymphoma: 5-year survival from the pivotal phase 2 CheckMate 205 study. Blood Adv (2023) 7(20):6266–74. doi: 10.1182/bloodadvances.2023010334 PMC10589773 37530622

[41] Kuruvilla J Ramchandren R Santoro A. Pembrolizumab versus brentuximab vedotin in relapsed or refractory classical hodgkin lymphoma (KEYNOTE-204): an interim analysis of a multicentre, randomised, open-label, phase 3 study. Lancet Oncol (2021) 22(4):512–24. doi: 10.1016/S1470-2045(21)00005-X 33721562

[42] Feng Y Hong Y Sun H. Abstract 2383: The molecular binding mechanism of tislelizumab, an investigational anti-PD-1 antibody, is differentiated from pembrolizumab and nivolumab. Cancer Res (2019) 79(13_Supplement):2383–3. doi: 10.1158/1538-7445.AM2019-2383

[43] Dahan R Sega E Engelhardt J. FcγRs modulate the anti-tumor activity of antibodies targeting the PD-1/PD-L1 axis. Cancer Cell (2015) 28(3):285–95. doi: 10.1016/j.ccell.2015.08.004 26373277

[44] Liu S-Y Wu Y-L. Tislelizumab: an investigational anti-PD-1 antibody for the treatment of advanced non-small cell lung cancer (NSCLC). Expert Opin Investig Drugs (2020) 29(12):1355–64. doi: 10.1080/13543784.2020.1833857 33044117

[45] Song Y Gao Q Zhang H. Tislelizumab for Relapsed/Refractory classical hodgkin lymphoma: 3-year follow-up and correlative biomarker analysis. Clin Cancer Res Off J Am Assoc Cancer Res (2022) 28(6):1147–56. doi: 10.1158/1078-0432.CCR-21-2023 PMC9365351 34716199

[46] Merryman RW Redd RA Nishihori T. Autologous stem cell transplantation after anti-PD-1 therapy for multiply relapsed or refractory hodgkin lymphoma. Blood Adv (2021) 5(6):1648–59. doi: 10.1182/bloodadvances.2020003556 PMC7993097 33710337

[47] Samara Y Mei M. Autologous stem cell transplantation in hodgkin lymphoma-latest advances in the era of novel therapies. Cancers (2022) 14(7):1738. doi: 10.3390/cancers14071738 35406509 PMC8996995

[48] Desai SH Spinner MA David K. Checkpoint inhibitor-based salvage regimens prior to autologous stem cell transplant improve event-free survival in relapsed/refractory classic hodgkin lymphoma. Am J Hematol (2023) 98(3):464–71. doi: 10.1002/ajh.26827 PMC11234511 36629030

[49] Carreau NA Armand P Merryman RW. Checkpoint blockade treatment sensitises relapsed/refractory non-hodgkin lymphoma to subsequent therapy. Br J Haematol (2020) 191(1):44–51. doi: 10.1111/bjh.16756 32430944

[50] Borcoman E Kamal M Marret G. HDAC inhibition to prime immune checkpoint inhibitors. Cancers (2021) 14(1):66. doi: 10.3390/cancers14010066 35008230 PMC8750966

[51] MeiMChenLGodfreyJ. Pembrolizumab plus vorinostat induces responses in patients with hodgkin lymphoma refractory to prior PD-1 blockade. Blood (2023) 142(16):1359–70. doi: 10.1182/blood.2023020485 37339586

[52] Hasenclever D Diehl V. A prognostic score for advanced hodgkin’s disease. international prognostic factors project on advanced hodgkin’s disease. N Engl J Med (1998) 339(21):1506–14. doi: 10.1056/NEJM199811193392104 9819449

[53] Hutchings M Loft A Hansen M. FDG-PET after two cycles of chemotherapy predicts treatment failure and progression-free survival in hodgkin lymphoma. Blood (2006) 107(1):52–9. doi: 10.1182/blood-2005-06-2252 16150944

[54] Gallamini A Hutchings M Rigacci L. Early interim 2-[18F]fluoro-2-deoxy-D-glucose positron emission tomography is prognostically superior to international prognostic score in advanced-stage hodgkin’s lymphoma: a report from a joint italian-danish study. J Clin Oncol Off J Am Soc Clin Oncol (2007) 25(24):3746–52. doi: 10.1200/JCO.2007.11.6525 17646666

[55] Bari A Marcheselli R Sacchi S. The classic prognostic factors in advanced hodgkin’s lymphoma patients are losing their meaning at the time of pet-guided treatments. Ann Hematol (2020) 99(2):277–82. doi: 10.1007/s00277-019-03893-7 PMC6976582 31872362

[56] Rodday AM Parsons SK Upshaw JN. The advanced-stage hodgkin lymphoma international prognostic index: Development and validation of a clinical prediction model from the HoLISTIC consortium. J Clin Oncol Off J Am Soc Clin Oncol (2023) 41(11):2076–86. doi: 10.1200/JCO.22.02473 PMC10082254 36495588

[57] Gallamini A Tarella C Viviani S. Early chemotherapy intensification with escalated BEACOPP in patients with advanced-stage hodgkin lymphoma with a positive interim positron emission Tomography/Computed tomography scan after two ABVD cycles: Long-term results of the GITIL/FIL HD 0607 trial. J Clin Oncol Off J Am Soc Clin Oncol (2018) 36(5):454–62. doi: 10.1200/JCO.2017.75.2543 29360414

[58] Stephens DM Li H Schöder H. Five-year follow-up of SWOG S0816: limitations and values of a PET-adapted approach with stage III/IV hodgkin lymphoma. Blood (2019) 134(15):1238–46. doi: 10.1182/blood.2019000719 PMC6788007 31331918

[59] Ricardi U Levis M Evangelista A. Role of radiotherapy to bulky sites of advanced hodgkin lymphoma treated with ABVD: final results of FIL HD0801 trial. Blood Adv (2021) 5(21):4504–14. doi: 10.1182/bloodadvances.2021005150 PMC8579271 34597375

[60] Johnson P Federico M Kirkwood A. Adapted treatment guided by interim PET-CT scan in advanced hodgkin’s lymphoma. N Engl J Med (2016) 374(25):2419–29. doi: 10.1056/NEJMoa1510093 PMC4961236 27332902

[61] Lang N Crump M. PET-adapted approaches to primary therapy for advanced hodgkin lymphoma. Ther Adv Hematol (2020) 11:2040620720914490. doi: 10.1177/2040620720914490 32537115 PMC7268111

[62] Sobesky S Mammadova L Cirillo M. In-depth cell-free DNA sequencing reveals genomic landscape of hodgkin’s lymphoma and facilitates ultrasensitive residual disease detection. Med (2021) 2(10):1171–1193.e11. 35590205 10.1016/j.medj.2021.09.002

[63] Spina V Bruscaggin A Cuccaro A. Circulating tumor DNA reveals genetics, clonal evolution, and residual disease in classical hodgkin lymphoma. Blood (2018) 131(22):2413–25. doi: 10.1182/blood-2017-11-812073 29449275

[64] Vandenberghe P Wlodarska I Tousseyn T. Non-invasive detection of genomic imbalances in Hodgkin/Reed-sternberg cells in early and advanced stage hodgkin’s lymphoma by sequencing of circulating cell-free DNA: a technical proof-of-principle study. Lancet Haematol (2015) 2(2):e55–65. doi: 10.1016/S2352-3026(14)00039-8 26687610

[65] Alig SK Esfahani MS Garofalo A. Distinct hodgkin lymphoma subtypes defined by noninvasive genomic profiling. Nature (2023). 10.1038/s41586-023-06903-x PMC11293530 38081297

[66] Kurtz DM Soo J Co Ting Keh L. Enhanced detection of minimal residual disease by targeted sequencing of phased variants in circulating tumor DNA. Nat Biotechnol (2021) 39(12):1537–47. doi: 10.1038/s41587-021-00981-w PMC8678141 34294911

[67] Kreissl S Goergen H Buehnen I. PET-guided eBEACOPP treatment of advanced-stage hodgkin lymphoma (HD18): follow-up analysis of an international, open-label, randomised, phase 3 trial. Lancet Haematol (2021) 8(6):e398–409. doi: 10.1016/S2352-3026(21)00101-0 34048679

[68] Borchmann P Goergen H Kobe C. PET-guided treatment in patients with advanced-stage hodgkin’s lymphoma (HD18): final results of an open-label, international, randomised phase 3 trial by the german hodgkin study group. Lancet Lond Engl (2017) 390(10114):2790–802. doi: 10.1016/S0140-6736(17)32134-7 29061295

[69] Borchmann P Haverkamp H Lohri A. Progression-free survival of early interim PET-positive patients with advanced stage hodgkin’s lymphoma treated with BEACOPPescalated alone or in combination with rituximab (HD18): an open-label, international, randomised phase 3 study by the german hodgkin study group. Lancet Oncol (2017) 18(4):454–63. doi: 10.1016/S1470-2045(17)30103-1 28236583

